# Functional alterations of the stomatognathic system in pacients with allergic rhinitis: case-control study

**DOI:** 10.1016/S1808-8694(15)30789-8

**Published:** 2015-10-19

**Authors:** Catiane Maçaira de Lemos, Niels Sales Willo Wilhelmsen, Olavo de Godoy Mion, João Ferreira de Mello

**Affiliations:** 1 Speech therapist, master's degree student, Division of the Otorhinolaryngological Unit of the Hospital das Clinicas da Faculdade de Medicina da USP; 2 Doctoral student, Division of the Otorhinolaryngological Unit of the Hospital das Clinicas da Faculdade de Medicina da Universidade de Sao Paulo. Voluntary dentist in the Stomatology Group of the Hospital das Clínicas da Faculdade de Medicina da Universidade de Sao Paulo; 3 Doctor in Otorhinolaryngology, Universidade de São Paulo. Collaborating professor, Universidade de Sao Paulo; 4 Livre-Docente (habilitation) professor, Faculdade de Medicina da Universidade de Sao Paulo. Assistant physician of the Hospital das Clinicas da Faculdade de Medicina da Universidade de Sao Paulo. Head of the Allergy in Otorhinolaryngology Group, Hospital das Clinicas da Faculdade de Medicina da Universidade de Sao Paulo

**Keywords:** deglutition, mastication, nasal obstruction, mouth breathing, stomatognathic system, articulation disorders

## Abstract

Mouth breathing can cause structural and functional alterations to the stomatognathic system.

**Aim:**

the aim of this investigation was to study breathing, chewing, swallowing and speaking alterations present in patients with allergic rhinitis and associate it to rhinitis symptom intensity.

**Materials and Methods:**

170 patients between the ages of 6 and 55 years were prospectively evaluated in this study, all of them underwent both otorhinolaryngological and speech evaluation.

**Results:**

the difference in signs and symptoms' score between GR and GC was significant.

**Conclusion:**

patients with allergic rhinitis have functional alterations in their stomatognathic system and an increase in nasal obstruction scores can be considered as a indication of such alterations.

## INTRODUCTION

Harmony in the human face acts as a mirror of expression and emotion, and is thus essential for speech and communication. The treatment of stomatognathic dysfunctions, therefore, should be part of public healthcare, given the implications for the integrated physiology of the mouth.^1^

Rehabilitation of stomatognathic dysfunctions is one of the aims of speech therapy in oromotor function.^2^

Nasal breathing is essential for the stomatognathic system to function normally, and for the maxillocraniofacial complex to grow and develop correctly.^3^

Individuals that for any reason acquire mouth or oronasal breathing patterns may compromise oral function and craniofacial, dental and phonoarticulatory organ development; in some cases other parts of the body may also be affected.^4^, ^5^, ^6^, ^7^, ^8^, ^9^, ^10^, ^11^, ^12^, ^13^, ^14^, ^15^, ^16^, ^17^, ^18^, ^19^, ^2^

The most frequent cause of mouth breathing is nasal and/or pharyngeal obstruction. Among the causes of nasal block, rhinitis has a high prevalence; some studies have suggested that its prevalence is gradually increasing.^13^^,^^14^

There are many types of rhinitis, which may be divided into two major groups: allergic and non-allergic. The latter may be subdivided into infections, non-allergic eosinophilic, idiopathic, irritative, gustative, and hormonal rhinitis, among others.^15^

Allergic rhinitis may be defined as an IgE-mediated nasal mucosa inflammation following exposure to antigens, and characterized by nasal block, pruritus, sneezing and coryza; at times, nasal block is the major symptom.^13^, ^14^, ^15^, ^16^, ^17^, ^18^, ^19^

According to the literature, there is a direct relation between rhinitis and nasal block, and of the latter with altered morphology and function of the stomatognathic system; few studies, however, have been conducted to observe these changes in rhinitis patients.^20^

Thus, the purpose of this study was to investigate the presence of changes in breathing, chewing, swallowing, and speech in patients with allergic rhinitis, and to relate these findings with the intensity of rhinitis symptoms.

### Series and Method

The Research Ethics Committee of the institution that is part of CONEP approved the research protocol (number 908/06) for this study.

A prospective study was undertaken of 170 male and female patients aged from 6 to 55 years. These patients were allocated to two groups:

• Rhinitis group (RG): 85 patients with a diagnosis of persistent allergic rhinitis (more than four days per week during more than four weeks).
• Control group (CG): 85 patients with no history, complaints or signs of nasal block of any etiology.

### Rhinitis group

For the rhinitis group (RG) all patients that visited for the first time an Allergy Outpatient Unit of a tertiary hospital from February to November 2006 were assessed. Based on the inclusion and exclusion criteria, 85 patients were selected for this group.

### Inclusion criteria - rhinitis group

Inclusion criteria for the RG were patients with a diagnosis of allergic rhinitis based on the clinical examination, the presence of signs and symptoms, and a personal and family history of atopy. The causative agent was confirmed by immediate hypersensitivity skin tests (puncture tests) for the relevant inhaled allergens in our context (Table 1).

**Table 1:** Antigens used in the immediate hypersensitivity skin tests

Group	Antigens
	*-Dermatophagoides pteronyssinus*
Acarids	*- Dermatophagoides farinae*
	*- Blomia tropicalis*
	*- Alternaria alternata*
Fungi	*- Cladosporium herbarum*
	*- Aspergillus fumigatus*
Cockroaches	*- Blatella germanica*
	*- Periplaneta americana*
Animal antigens	*- Canis familiaris*
	*- Felis domesticus*
	- Phleum pratense
Pollens	*- Lolium perenne*
	*- Dactylis glomerata*
	*- Festuca pratensis*

### Exclusion criteria - rhinitis group

patients with a history or cavum radiographs demonstrating a condition other than allergic rhinitis that has nasal block as an associated symptom;

patients with neurological, neuromuscular, motor or skeletal alterations;

patients undergoing speech therapy.

### Control group

The control group (CG) was formed in two steps: first of all, a 4-question questionnaire was applied to investigate the presence of complaints associated with the symptoms of allergic rhinitis (Annex 1). Participants that answered NO to all of the questions underwent an otorhinolaryngological evaluation to discard the presence of nasal and/or pharyngeal obstruction.

### Inclusion criteria - control group

Subjects with no complaints, signs or symptoms of rhinitis or other conditions that have nasal block as a symptom were included in this group.

### Exclusion criteria

patients with neurological, neuromuscular, motor or skeletal alterations;

patients undergoing speech therapy.

Patients in both groups were subdivided into three age groups: children – 6 to 11 years, adolescents – 12 to 18 years, and adults – 19 to 55 years.

Each patient underwent a clinical assessment based on the recognized Signs and Symptoms Score.^15^^,^^16^ (Table 2)

**Table 2:** Nasal signs and symptoms score

Symptoms	Signs
**Sneezing / pruritus**	**Color of nasal turbinates**
0- Absent	0- Pink
1– 1 to 4 per day / occasional pruritus	1– Reddened / light pink
2– 5 to 10 per day / sporadic pruritus for 30 minutes	2– Red / light
3– 11 or more / interferes with sleep and/or concentration	3– Anemic / bluish
**Coryza**	**Edema nasal turbinates**
0- Absent	0- Absent
1- Cleaning 1 to 4 times a day	1- Hypertrophic lower or middle turbinate with minor nasal block
2- Cleaning 5 to 10 times a day	2- Congested nose affecting breathing in one of both nasal fossae
3- Constant cleaning	3- Congestion hindering breathing in one or both nasal fossae
**Nasal block**	**Secretion**
0- Absent	0- Absent
1- Minor, not bothersome	1- Mucosa appears humid
2- Mouth breathing most of the day	2- Visible secretion on turbinates or floor of the nasal fossa
3- No nasal breathing / interferes with sleep, olfaction or voice	3- Profuse / draining
**Retronasal secretion**	**Posterior wall of oropharynx**
0- Absent	0- Normal
1- Feeling of secretion in throat	1- Mildly red
2- Frequent cleaning of throat	2- Hyperemic / apparent lymphoid follicles
3- Coughing / affecting speech	3- Visible mucus

All patients underwent a Phonoaudiological assessment; the same speech therapist evaluated breathing, chewing, swallowing and speech oral functions.

The breathing mode was defined as follows:

1 normal: if noted and reported by patients/caretaker as being nasal (daily and nightly).
2 altered: in cases different from the description above.

Patients were asked to chew a biscuit, to assess chewing function. Based on this observation, chewing was defined as:

1 normal: if done bilaterally with the lips closed.
2 altered: in cases different from the description above.

The swallowing pattern was observed in this study by direct observation of swallowing water from a cup, as follows:

1 normal: if done with the lips closed, the tongue positioned on the palatine papilla, and without including the periorbicular muscles.
2 altered: in cases different from the description above.

The articulatory pattern was noted based on naming of figures (BEFFI, 2000), and was classified as follows:

1 normal: when there were no phoneme changes, omissions and/or distortions.
2 altered: in cases different from the description above.

### Statistics

Student's T test was applied to verify the age similarities among the groups.

The verosimilarity ratio test was applied to compare the scores of the otorhinolaryngological evaluation among the study and control groups.

Data on the breathing mode, chewing function and swallowing and articulatory patterns were compared among groups at different age ranges. The verosimilarity ratio test was applied to check whether there was any difference in the distribution of variables for each function in both groups and in the different age ranges.

The Mann-Whitney test was applied to verify the presence of any correlation among obstruction scores and altered function.

## RESULTS

The mean age of 85 patients in the RG was 7.6 years (+/− 2.3 years) in children, 13.2 years (+/− 1.6 years) in adolescents, and 29.2 years (+/− 10.2 years) in adults. The mean age in the CG was 7.3 years (+/− 2.2 years) in children, 14.6 years (+/− 2.0 years) in adolescents, and 30.4 years (+/− 9.7 years) in adults.

Tables 3 and 4 show the sex distribution in both groups.

**Table 3:** Sex distribution - Rhinitis group

	Children	Adolescents	Adults	Total
	N (%)	N (%)	N (%)	N (%)
Female	11 36,7	13 43,3	21 84,0	49 57.6
Male	19 63,3	17 56,7	4 16,0	36 42.4
Total	30 100	30 100	25 100	85 100

**Table 4:** Sex distribution - Control group

	Children	Adolescents	Adults	Total
	N (%)	N (%)	N (%)	N (%)
Female	15 50	18 60	15 60	48 56.5
Male	15 50	12 40	10 40	37 43.5
Total	30 100	30 100	25 100	85 100

Table 5 shows the data comparing the otorhinolaryngological evaluation scores in the rhinitis group and the control group.

**Table 5:** Distribution of scores

		Median	Minimum	Maximum	
	CG	1,6	0	3	p <
Score of signs					
	RG	5,1	1	9	0,001*
Score of symptoms	CG	0,7	0	3	p <
	RG	4,9	0	10	0,001*
Obstruction score	CG	0,2	0	1	p <
	RG	1,6	0	3	0,001*
Total score (signs + symptoms)	CG	2,3	0	5	p <
	RG	9,9	1	19	0,001*

Chart I, Chart II, Chart III, Chart IV show the distribution of variables of each orofacial function in both groups and three age ranges.

**Chart I:**
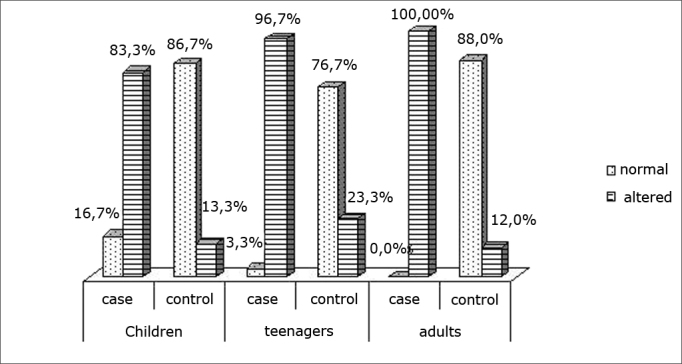
Distribution of the breathing mode

**Chart II:**
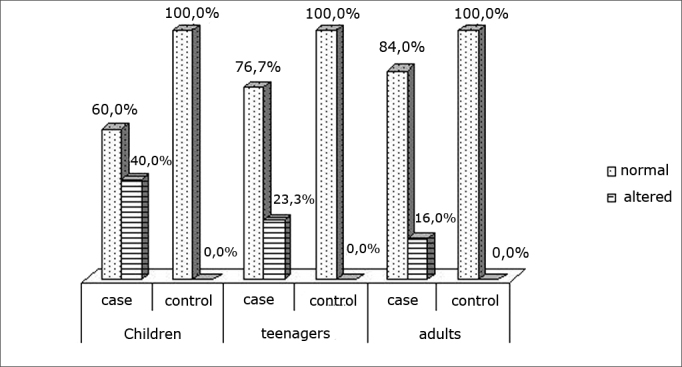
Distribution of the chewing pattern

**Chart III:**
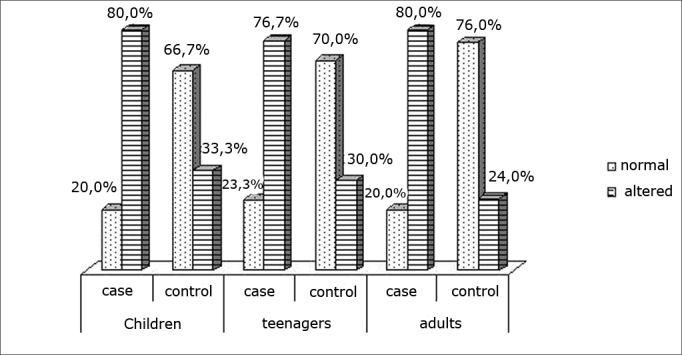
Distribution of the swallowing pattern

**Chart IV:**
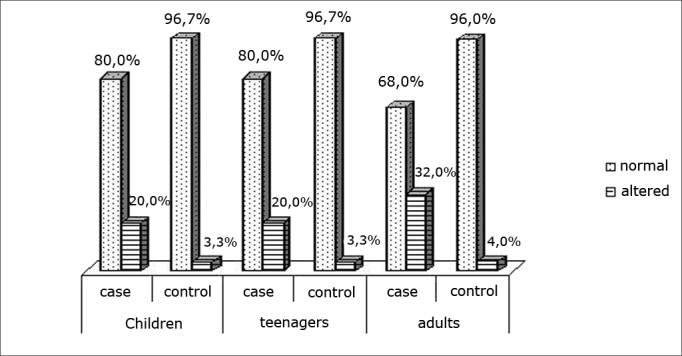
Distribution of the articulatory pattern

Table 6 shows the analysis of the correlation between an increased obstruction score and altered function in the RG

**Tabela 6:** Correlação entre escore obstrução e alteração funcional

	Modo respiratório	Padrão mastigatório	Padrão de deglutição	Padrão articulatório
Escore Obstrução	p < 0,001*	p = 0,039*	p = 0,267	p = 0,80

## DISCUSSION

The sex distribution of the sample (Table 3, Table 4) shows that children and adolescent in the RG were mostly male. Di Francesco et al. 2004^9^ found a similar distribution in a study of 142 mouth-breathing patients aged 2 to 16 years; Marques et al.^10^ reported a similar result. Among adults, however, there were no studies with this age distribution; in this case we believe that a smaller number of males may be explained by the fact that men attribute less importance to allergic rhinitis symptoms and have less time to seek ambulatory medical care.

Because rhinitis is defined clinically as the sum of various signs and symptoms, observing and measuring these findings is important in medical practice.^14^^,^^15^ In this study we found a statistically significant difference between the rhinitis and control groups in the comparison of scores for signs and symptoms and total scores. (Table 5). These findings underline the applicability of these tests in a clinical setting, as confirmed in the literature.^14^^,^^15^

A specific analysis was made of the nasal block score, because this symptom predominates in allergic rhinitis^14^, ^15^, ^16^, ^17^, ^18^ and it relates directly with the presence of dysfunctions in the stomatognathic system^4^^,^^6^^,^^8^^,^^9^^,^^11^^,^^12^^,^^21^^,^^22^. Table 5 shows the comparison of this symptom in both groups, which revealed a statistically significant difference.

In the analysis of changes in the stomatognathic system we found a high rate of altered breathing in rhinitis group patients (Chart I); this percentage was significantly higher than that found in the control group at all ages. Barros et al. 2003^23^ also found similar results in a study of 140 mouth-breathing patients; among these patients 44.3% were positive in allergic tests.

Many studies have reported the clinical implications and orofacial changes in mouth-breathing patients. Our finding of a high rate of altered breathing in our sample suggested that there was a high probability of findings other stomatognathic dysfunctions.

Chewing is a learnt function, and may undergo changes. The first dentition has to be fully developed for an individual to be able to chew. We found that chewing function alterations decreased with age (Chart II). We believe that although all patients were aged over 6 years (complete deciduous dentition), this reduction may have been due to the maturity of the chewing process.^24^ However, there were still statistically significant differences among the rhinitis and the control group at all ages.

We did not find any studies in the literature on chewing performance in adolescent or adult allergic rhinitis patients. However, our results are similar to those in studies done in mouth-breathing children.^4^^,^^25^ One such study of 46 children with a deciduous dentition found a statistically significant difference in the position of open or closed lips during chewing between nasal breathing and mouth breathing children.^25^ Another study of patients with adenoid and tonsillar hypertrophy found altered chewing patters in 88.5% of the sample.^4^

The age at which swallowing becomes mature is a controversial issue in the literature; estimates range from 18 months to 6 years.^26^ By these estimates, all patients in our study had already reached the mature phase of swallowing. Many studies have pointed out the relation between mouth breathing and the presence of changes in swallowing patterns;^4^^,^^27^^,^^28^ however, we found no published papers analyzing this dysfunction in allergic rhinitis patients.

We found a statistically higher percentage of patients with altered swallowing patterns in the RG compared to the CG at all three age ranges; this shows that such alterations result from changes in air flow, since none of these patients were in a transition phase for developing this function. We found, even in the control group, many patients with dysfunctional swallowing. This may be explained by dysfunctional occlusion or altered facial typology, as demonstrated in published papers;^27^, ^28^, ^29^, ^30^ these findings are the objects of another paper still to be published.

The assessment of joints among the groups and ages showed no statistically significant differences. We found no published papers that correlated altered speech with altered breathing. However, some studies have found dysfunctional articulations in patients with occlusion disorders due to mouth breathing,^31^^,^^32^ showing that in most cases, altered breathing needs to be accompanied by malocclusion for there to be speech disorders.

The analysis of changes in function revealed a correlation between an increased nasal block score (otorhinolaryngological evaluation) and the presence of altered function (Table 6). We found a significant correlation between the breathing mode and chewing function. These data confirm the findings in the literature that such changes may occur as a consequence of obstruction.^4^^,^^25^ Altered swallowing and articulation function did not correlate with increased nasal block scores, and may occur regardless of nasal obstruction. These data diverge from other reports in the literature.^3^^,^^5^^,^^7^^,^^12^

## CONCLUSION

Our analysis of data in this study revealed that: allergic rhinitis patients present altered breathing, chewing and swallowing.

Increased nasal obstruction scores may be considered as indicating the presence of the abovementioned changes.
